# Noise spectrum characteristics of marine pump units induced by different excitation sources

**DOI:** 10.1038/s41598-022-12755-8

**Published:** 2022-05-23

**Authors:** Houlin Liu, Runze Zhou, Qi Pan, Liang Dong, Qijiang Ma, ZhiMing Cheng, Xiaolin Wang

**Affiliations:** 1 National Research Center of Pumps, Jiangsu University, Zhenjiang, 212013 China; 2 Research Center of Fluid Machinery Engineering and Technology, Jiangsu University, Zhenjiang, 212013 China

**Keywords:** Energy science and technology, Engineering

## Abstract

To study the noise spectrum characteristics of marine pump units induced by different excitation sources, a computational aeroacoustic (CAA) model of the internal and external field noise of a marine pump was established. The coupled acoustic-vibration method was used to obtain the spectrum characteristics of internal and external field noise. The accuracy and feasibility of the simulation method for noise prediction were confirmed through a noise test. Due to the different mediums in the internal and external fields of the marine pump, an external field acoustic model was established based on the automatically matched layer (AML) technology. The spectral characteristics of different excitation sources and the spatial distribution of the radiated sound field were analyzed, and the contribution of different sound source excitations to the internal and external sound field was revealed. The results show that the main frequency of the internal field noise generated by different excitations is at the blade passing frequency, and the internal field noise induced by the dipole acoustic excitations dominates at 180.6 dB. For the external field noise, the main frequency is still located at the blade passing frequency. The radiation noise induced by the fluid excitation (139.2 dB) is higher than that induced by the dipole excitations (surface dipole, 136.3 dB; rotating dipole, 137.3 dB).

## Introduction

Marine centrifugal pumps are essential auxiliary equipment on ships and play a vital role in the regular operation of ships. Marine pumps produce loud noise during operation, and the noise generation mechanism is complex. The noise level of marine pumps is critical, especially for military vessels. Vibration and noise are inseparable during pump operation. Vibration generates noise, and noise also affects vibration. There are many noise sources for pump units, and the most common source is the noise caused by the vibration of the pump units, which is structural vibration noise. The noise generated by fluid flow is called hydrodynamic noise^1–4^, which makes a greater contribution to the noise of the pump units, and the generation mechanism is also complicated.

The concept of hydrodynamic noise was initially developed due to the Lighthill acoustic analogy theory^5^. Subsequently, Williams and Hawkings^6^ applied the governing equation to the boundary problem of solid motion and proposed the famous FW-H equation to divide hydrodynamic noise sources into monopole, dipole and quadrupole sound sources. For ease of comprehension, scholars classified noise into broadband noise and discrete noise, namely, single-tone noise^7^. Among the flow-induced noise of the pump, monopole source noise is induced by the volume compression effect of the pump cavitation and is a discrete noise. The dipole source is mainly caused by the fluid's unsteady fluctuating force acting on the structure's surface, which includes broadband noise and discrete noise. The quadrupole source is caused by the turbulence generated by the high-speed fluid flow, which is classified as broadband noise^8^.

Dong et al.^9^ studied the pressure pulsation and radial force characteristics in the unsteady flow process at different cavitation stages through tests. Howe^10,11^ pointed out that the main sound source of rotating machinery is the dipole sound source caused by the unsteady force and proposed that the flow field can be solved first and then the sound field can be further solved according to the obtained flow field results. Zhou et al.^12^ proposed that the sources of hydrodynamic noise are similar when the fluid Reynolds numbers are similar. The main acoustic calculations for the internal and external fields of pumps are the boundary element method (BEM) and the finite element method (FEM). Si et al.^13^ used the direct BEM to calculate the sound field in a centrifugal pump and found that the blade passing frequency and multiplier are the characteristic frequencies of the fluid-induced noise. Cai et al.^14^ and Yu et al.^15^ also used this method to calculate the internal noise of submersible sewage pumps and vortex self-priming pumps. Allen^16^ proposed the coupled FEM/BEM to calculate the structure radiated noise by the boundary layer, using fluctuating pressure on the wall to define the excitation on the structure-acoustic system. Warszawski et al.^17^ used the FEM/BEM to study the propagation characteristics of acoustic waves generated by fluid–structure interactions. Han et al.^18^ realized the acoustic-vibration coupling calculation for the con-shell structure by the FEM/BEM, obtained the sound pressure level at the measuring point, and verified the accuracy of the calculation result through experiments. Liu et al.^19^ studied the influence of the blade outlet angle and the width on the fluid-induced noise of the centrifugal pump by the direct BEM method. Dai et al.^20^ also calculated the external field noise of a centrifugal pump by coupled acoustic vibration.

Although the mediums are different inside and outside the marine pump, the boundary element can define only one fluid property, so this method cannot effectively solve the noise problem in the external field of the pump. In contrast, the acoustic finite element method is effective to calculate radiation acoustics. Xie et al.^21,22^ predicted the underwater low-frequency noise of a small-scale stiffened shell based on the acoustic finite element method and proved the effectiveness in predicting the infinite sound field. Majda^23^ proposed the absorption boundary condition, which replaces the infinite external sound field with a convex fluid domain. The sound absorption boundary condition is imposed on the convex fluid domain. Berenger^24^ used the perfectly matched layer (PML) method to calculate the fluctuation of the electromagnetic field. Because this method requires experienced engineers to complete the division of the absorption layer, the automatically matched layer (AML) technology is proposed. The AML method automatically generates matching layer cell domains based on the structural finite element area, and the size of the cell domain varies according to the analysis frequency, which can improve the calculation efficiency.

There have been few studies on the effects of fluid excitation and dipole excitations on pump noise, and the primary mechanism of pump noise generation is unclear. Therefore, according to the generation and transmission mode of the noise for marine pump units, the present paper established a computational aero-acoustic model for the internal and external radiated noise generated by fluid flow. Considering the coupled structure-sound field, the acoustic finite element method was used to calculate the internal field noise under fluid excitation and dipole acoustic excitations. The accuracy and feasibility of the coupled acoustic-vibration method for predicting internal field noise was confirmed by test comparison. Due to the different mediums in marine pumps' internal and external fields, an external acoustic model based on AML technology was established. The spectral characteristics of different noise sources and the spatial distribution of the radiated sound field were analyzed, and the contributions of different sound sources to the external sound field were revealed.

## Numerical calculation model

A marine centrifugal pump with a specific speed of 66.7 was used as the research object. The flow rate *Q*_d_ = 25 m^3^/h, head *H* = 35 m, rotational speed *n* = 2950 r/min. Table 1 shows the main geometric parameters of the marine pump.

**Table 1:** Main geometric parameters of the marine pump.

Components	Geometric parameters	Symbol	Value
Impeller	Inlet diameter	*D*_*1*_	65 mm
	Outlet diameter	*D*_2_	165 mm
	Outlet width	*b*_2_	7 mm
	Blade wrap angle	*φ*	110°
	Blade numbers	*z*	6
Volute	Inlet diameter	*D*_3_	170 mm
	Inlet width	*B*_*3*_	20 mm
	Outlet diameter	*D*_d_	50 mm

**Figure 1:**
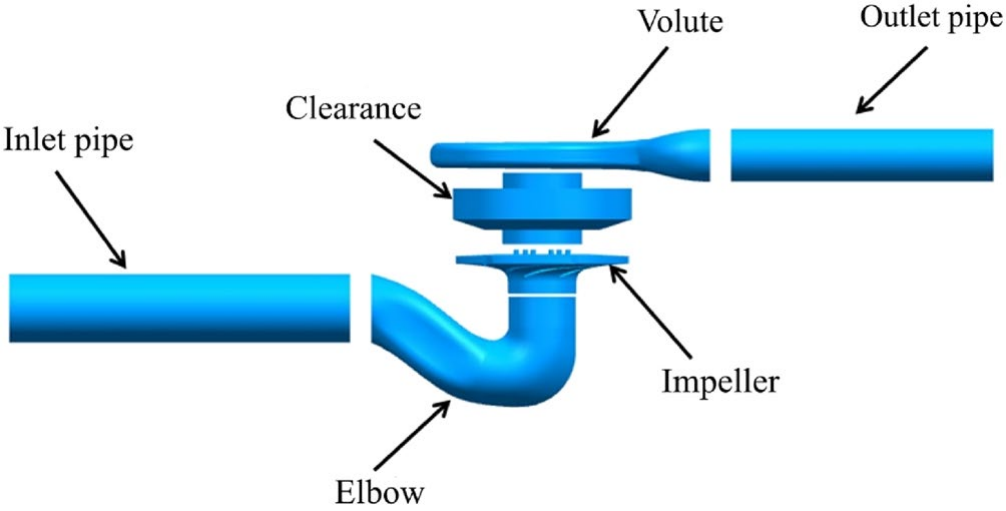
Entire flow field model.

**Figure 2:**
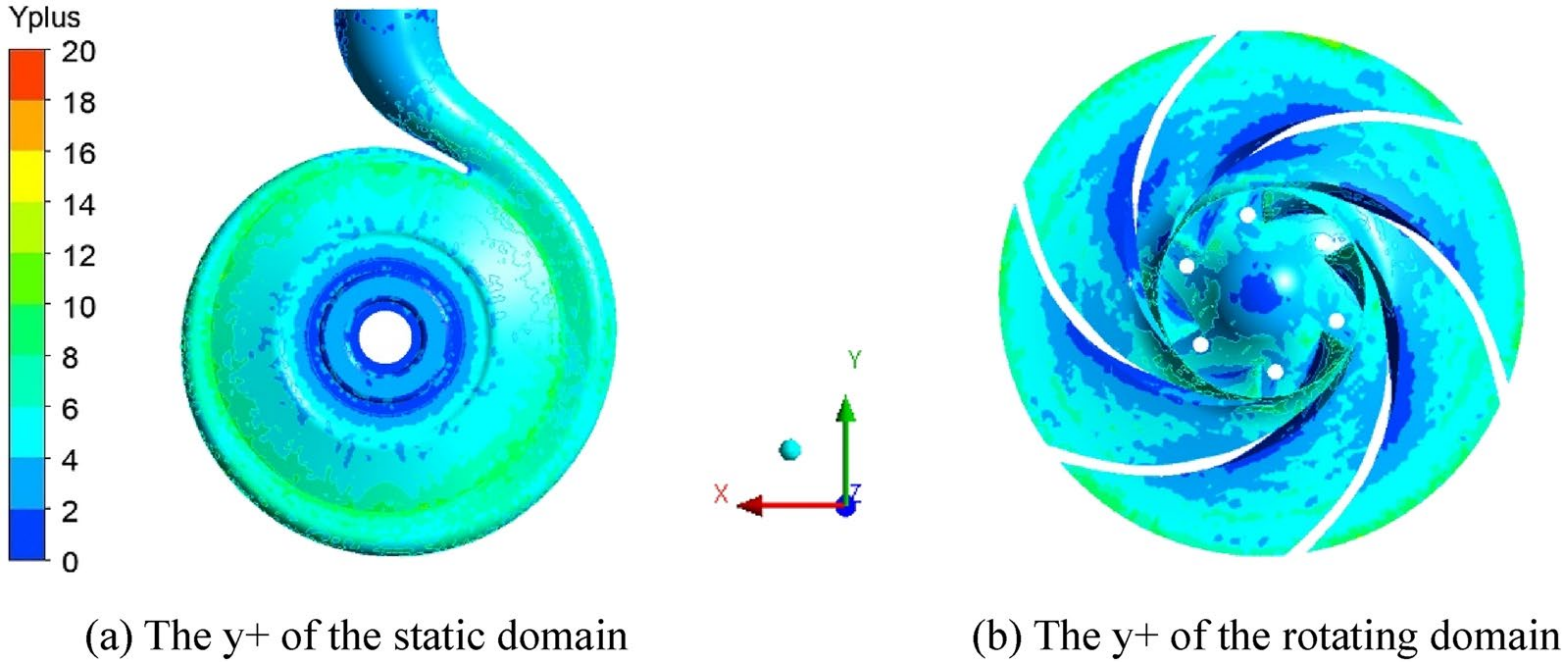
The y + distribution at the volute and the pump chamber and the impeller.

The entire flow field calculation model includes the inlet pipe, elbow, impeller, clearance (between the impeller and the volute), volute and outlet pipe, as shown in Fig. 1. ANSYS ICEM 17.0 was used to generate the fluid domain grid. Table 2 shows the grid-independence verification. The error of the head is less than 1% in the second case, so this case was selected for subsequent calculations. Figure 2 shows the wall y + of the static domain and the rotating domain of the model pump. The average of y + is less than 12. The y + in the static domain is less than 6, while the y + of the inner impeller wall is less than 8. The turbulence model used the standard *k-ε* model, and pressure-inlet and mass-flow-outlet conditions were adopted. The interface between the domains used the general grid interface (GGI). The convection term adopts the second-order upwind. The time step was set to ΔT = 0.565 × 10^–4^ s, i.e., every 1° rotation of the impeller. The pressure pulsation data of 10 stable cycles of impeller rotation were extracted as the excitation source, and the total time was set to 0.25 s. The residual was set as 10^–4^.

**Table 2:** Mesh-dependence verification.

Case	Number of elements	Number of nodes	Head/m
1	1,647,157	1,474,148	34.5
2	2,457,849	2,287,414	35.2
3	2,914,979	2,741,943	35.5
4	3,278,458	3,024,785	35.5
5	3,715,756	3,546,854	35.6

## Internal field noise

### Acoustic simulation method

LMS Virtual.Lab 13.6 software was used to calculate the internal field noise of the marine pump. The noise of the marine pump can be divided into (1) flow-induced noise, which is the fluid excitation source radiating sound to the structure, causing the structure to vibrate and thus generating radiated noise, and (2) flow noise, which is directly radiated by the dipole excitation source. Moreover, there are two primary dipole excitation sources for the pump: a rotating dipole excitation on the impeller surface and a surface dipole excitation on the volute wall.

For the calculation of flow-induced noise, the pressure pulsation action on the pump wall was used as the excitation. The data on the cell nodes were transferred to the grid of the pump structural model, and the coupled acoustic-vibration algorithm was used to calculate the structural vibration of the pump and obtain the flow-induced noise. For the noise induced by the surface dipole, the pulsation of the pump wall was first used as the boundary condition of the acoustic dipole. Then, the geometric interpolation method without energy loss was used to transfer the fluid field information to the acoustic grid, the sound pressure on the inner wall was calculated, and the noise induced by the surface dipole was calculated. For the noise induced by the rotating dipole on blades, the time-domain pulsation data on the blade were extracted, and the discrete blade source was used to calculate the sound field. The impeller was divided into four parts, and the load and its acting position are defined on each part. The sound pressure on the wall of the internal field pump was calculated, and then this result was used as the acoustic excitation for the coupled acoustic-vibration calculation to obtain the noise excited by the rotating dipole.

**Figure 3:**
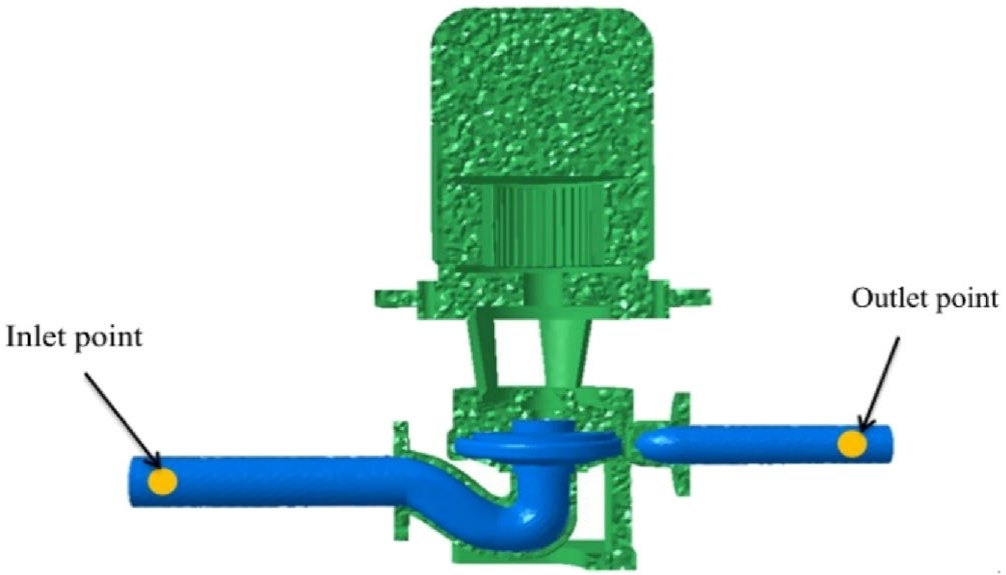
Acoustic-vibration model and monitoring points.

The inlet and outlet of the infield acoustic model were defined as full sound absorption properties, and the remaining surfaces were defined as full reflection walls. The characteristic acoustic impedance *Z* = *ρc* = 1.5 × 10^6^ kg/(m^2^·s), and the speed of sound was taken as 1500 m/s. The internal field acoustic-vibration model and the locations of the inlet and outlet monitoring points are shown in Fig. 3. The sound pressure level is the most commonly used indicator of acoustic wave intensity. It correlates well with the human perception of loudness and is expressed as follows:

1 $$ SPL = 20\lg (p/p_{ref} ) $$

where *p* is the sound pressure and *p*_ref_ is the reference value of the underwater sound pressure, 1 μPa.

### Acoustic modal calculation

**Figure 4:**
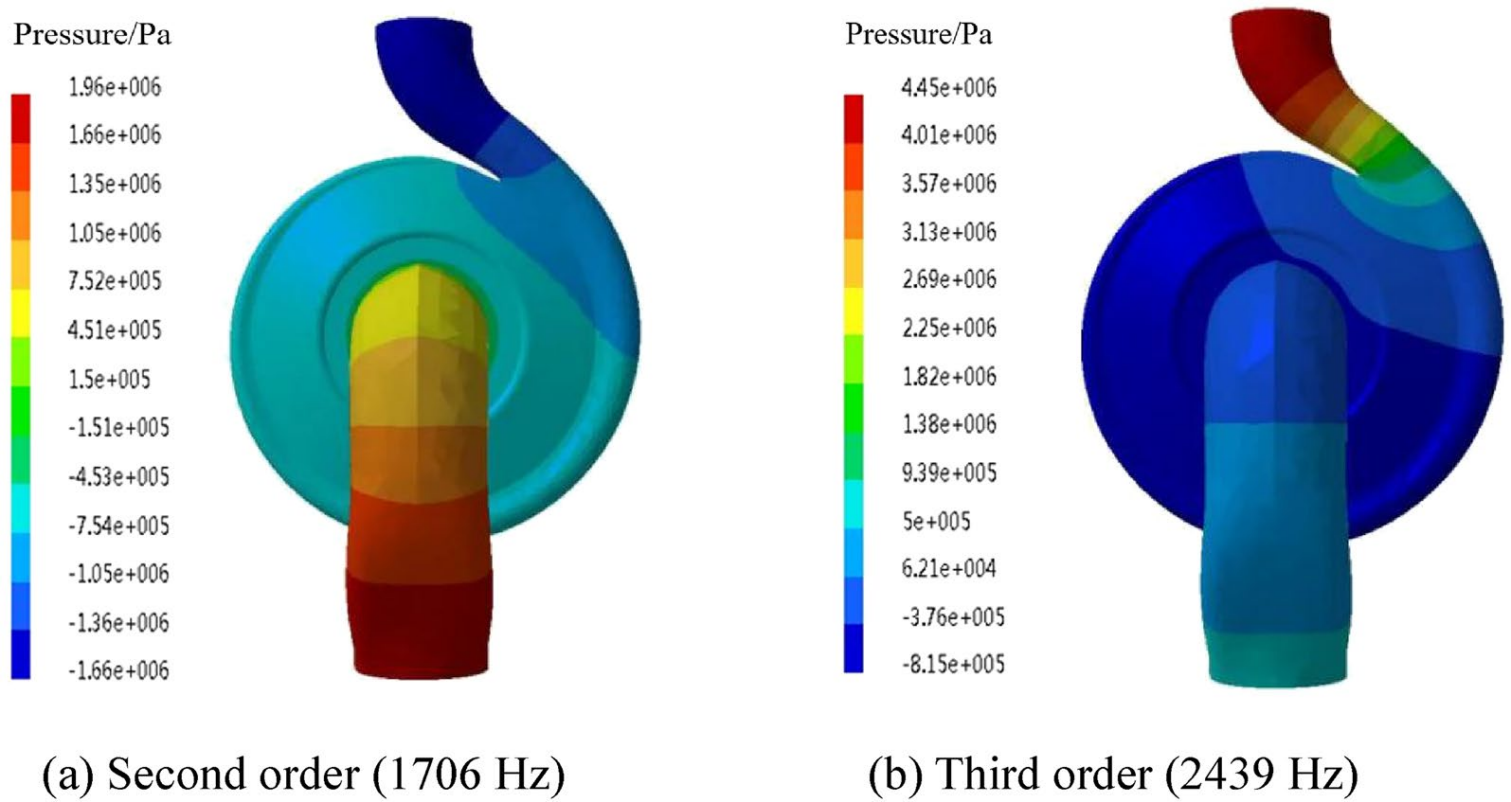
Internal acoustic mode.

The coupled acoustic-vibration calculation of the marine pump is carried out based on the modal space. It is necessary to comprehensively consider the structural and acoustic modes to calculate the internal field noise accurately. The internal acoustic mode of the marine pump is shown in Fig. 4. Since the acoustic unit has only 1 degree of freedom in space and the sound field model is in a free state, its first-order mode is a rigid body mode, and its natural frequency is 0 Hz. The second-order mode shape indicates that the high-sound-pressure region is concentrated in the inlet pipe and the volute diffuser but in opposite phases. There is a gradient drop from the inlet to the outlet. The third-order mode shape has the highest sound pressure in the outlet, and the inlet pipe has a lower sound pressure. Moreover, the volute is in the opposite phase to the other positions.

### Internal field noise by fluid excitation

**Figure 5:**
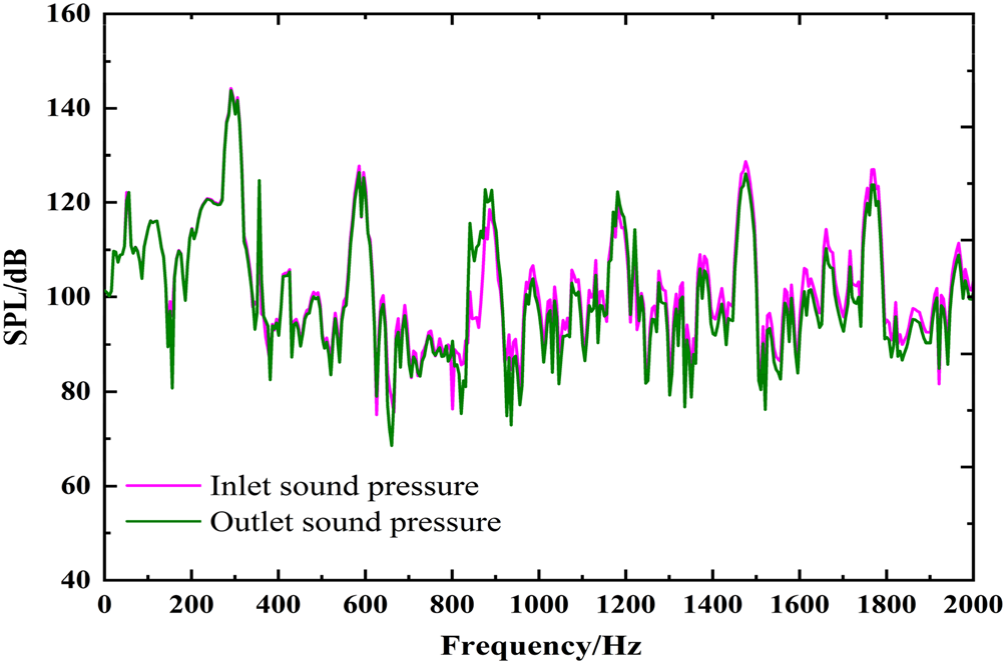
The spectrum of infield noise by fluid excitation.

Figure 5 shows the broadband spectrum of the internal field noise induced by fluid excitation. The sound pressure level spectrum trends are generally consistent for the inlet and outlet. The main frequency is located at the blade passing frequency (BPF), and the harmonic frequencies are also at each multiple blade passing frequency. This phenomenon is mainly caused by rotor–stator interference when the blade sweeps through the volute tongue. The sound pressure level at the blade passing frequency is as high as 144 dB. Several peaks appear at the characteristic frequencies, and the characteristics of the main frequency and harmonic frequencies are not prominent.

By comparing the inlet and outlet sound pressure spectra, it can be found that the law and peak values are consistent from 0 to 590 Hz (2BPF). The inlet and outlet sound pressure spectra are almost identical at the twice blade passing frequency and subsequent frequency bands, but the broadband peak is different. Overall, below 1000 Hz, the sound pressure level at the outlet is higher at rated operating conditions. However, the inlet sound pressure level is higher at some special frequencies between 1000 and 2000 Hz.

### Internal field noise by dipole excitations

#### Surface dipole sound source

**Figure 6:**
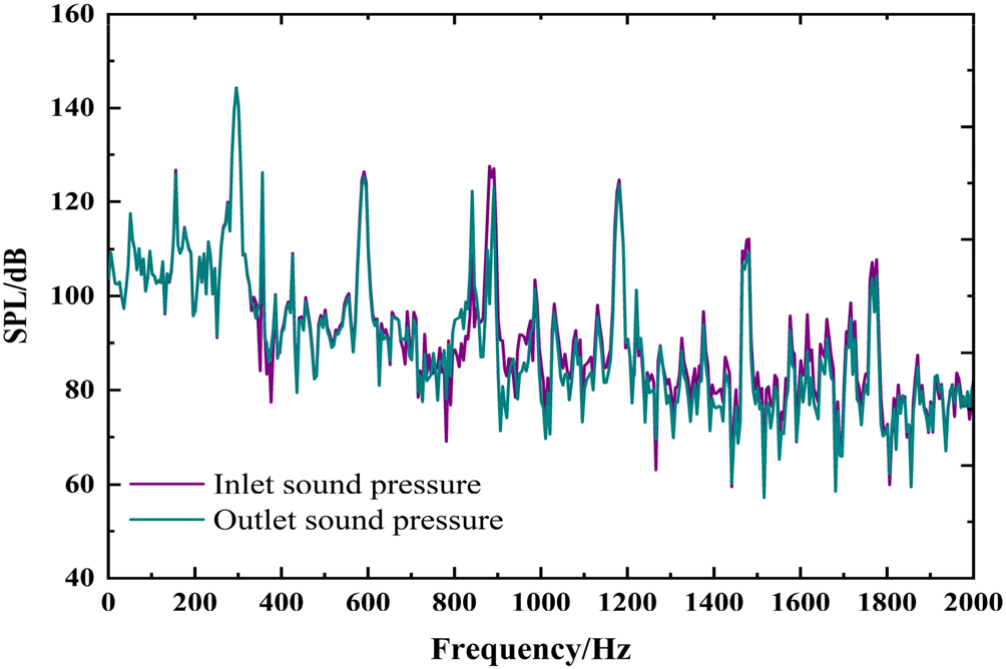
The spectrum of infield noise by surface dipole.

Figure 6 shows the broadband spectrum of internal field noise induced by the surface dipole. The trends of the sound pressure level at the inlet and outlet are almost the same; the main frequency appears at the blade passing frequency, and the harmonic frequency is also at each multiple blade passing frequency. The maximum sound pressure level reaches 149.8 dB. The internal field noise generated by the surface dipole appears as single distinct peaks at the characteristic frequencies. The average sound pressure at the outlet is higher than that at the inlet, but the inlet and outlet sound pressure levels are similar due to the conversion by logarithms.

#### Rotating dipole sound source

**Figure 7:**
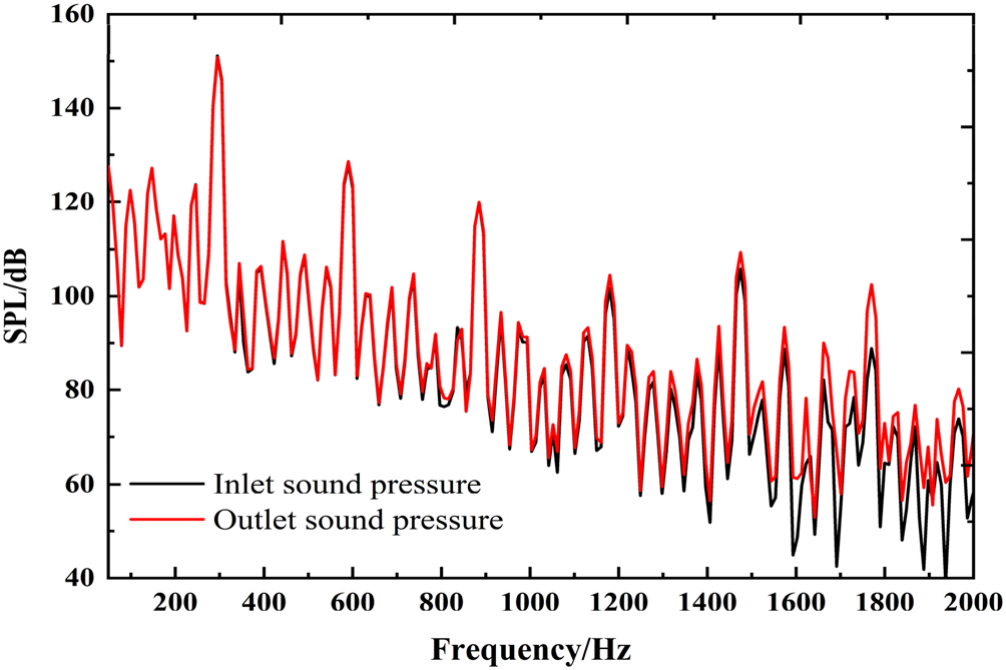
The spectrum of infield noise by the rotating dipole.

The finite element method is used to calculate the internal sound field coupled with the acoustic excitation of the rotating dipole, as shown in Fig. 7. The frequency response trends of inlet and outlet sound pressure levels are also similar. The main frequency appears at the blade passing frequency, and the harmonic frequency is also at each multiple blade passing frequency. The internal field noise by the rotating dipole is more evident than the surface dipole in the harmonic characteristics of each characteristic frequency. Moreover, the peaks at each blade passing frequency are more apparent than those at other frequencies. The sound pressure level at the blade passing frequency reaches 150 dB, indicating that the rotating dipole greatly influences the internal field noise at the characteristic frequency.

**Figure 8:**
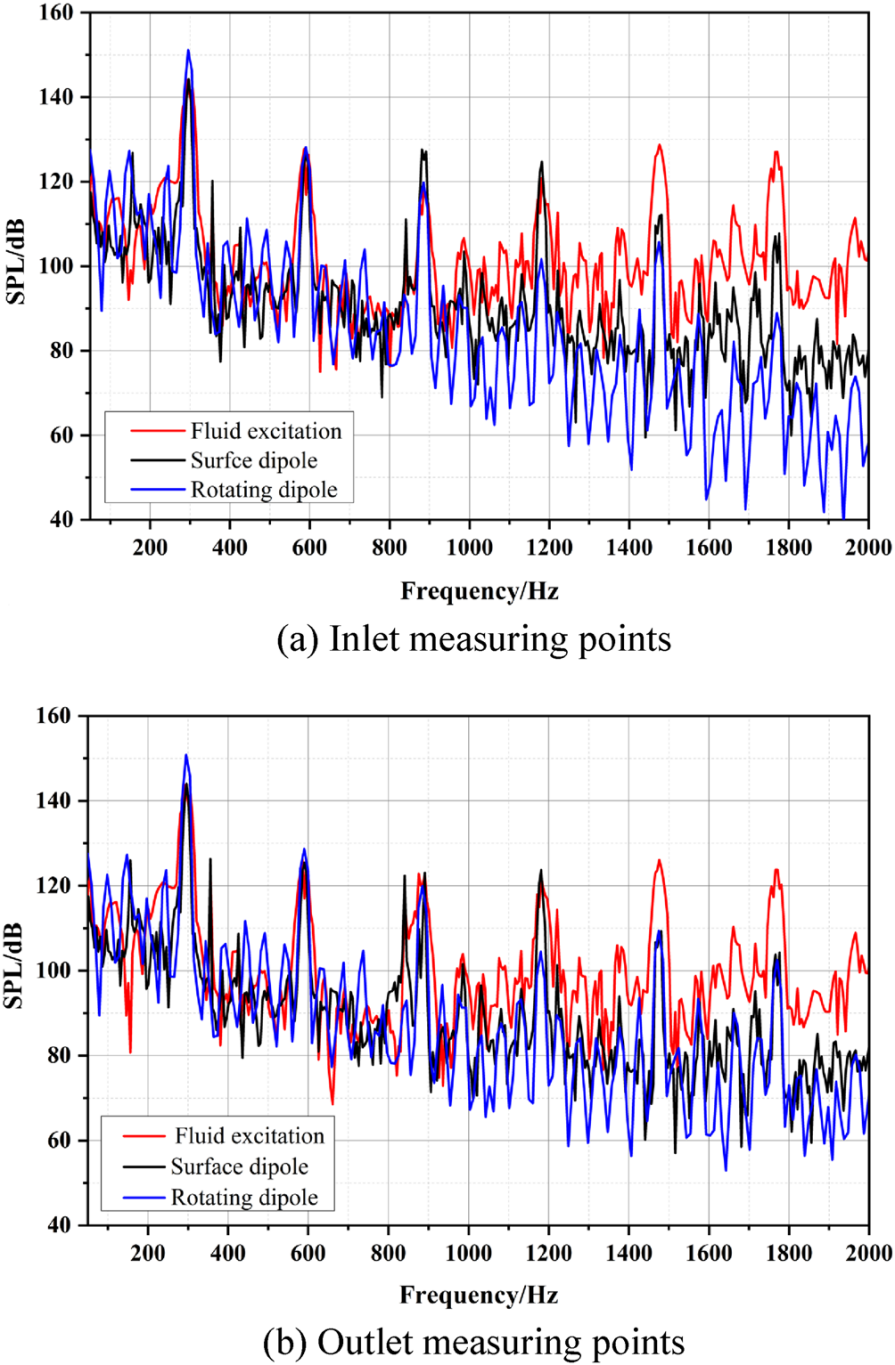
The spectrum of infield noise by different excitations.

The spectral curves of the field noise at the inlet and outlet induced by fluid excitation and acoustic dipoles are shown in Fig. 8. The rotating dipole sound pressure level is the highest at the BPF and two times the BPF (abbreviated as 2BPF). However, the average sound pressure level of the surface dipole is higher below 1000 Hz. The internal field noise induced by fluid excitation presents a higher level in the entire frequency band between 1000 and 2000 Hz. The sound pressure of the noise caused by the rotating dipole is relatively low, and this characteristic is particularly prominent at the outlet.

**Figure 9:**
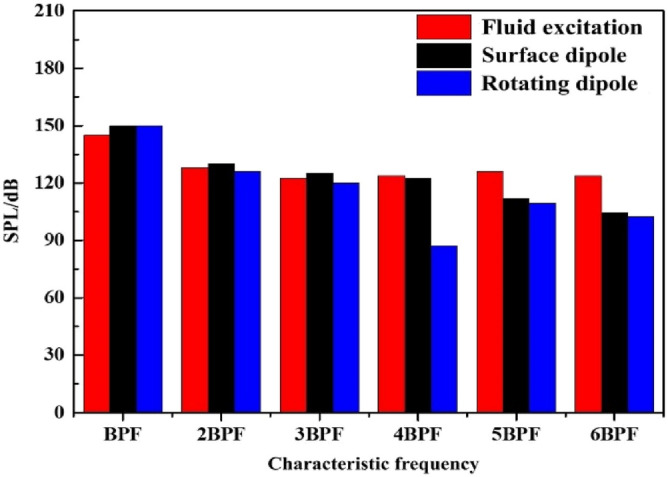
Sound pressure level at each characteristic frequency of different excitations.

Figure 9 shows the sound pressure levels at each characteristic frequency of different excitations. The sound pressure level of the internal field noise induced by the rotating dipole at the blade passing frequency (BPF = 295 Hz) is close to that of the surface dipole, 150 dB and 149.8 dB, respectively. The flow-induced noise contributes the least at the BPF, with a sound pressure level of only 144 dB. At 2BPF (590 Hz), the peaks of the three excitation sources are relatively close, and the internal field noise caused by the surface dipole is slightly higher, at 128 dB. When exceeding 4BPF, the fluid excitation gradually dominates, especially at 5BPF and 6BPF, and the proportion of dipole excitations decreases.

## Internal field noise test

**Figure 10:**
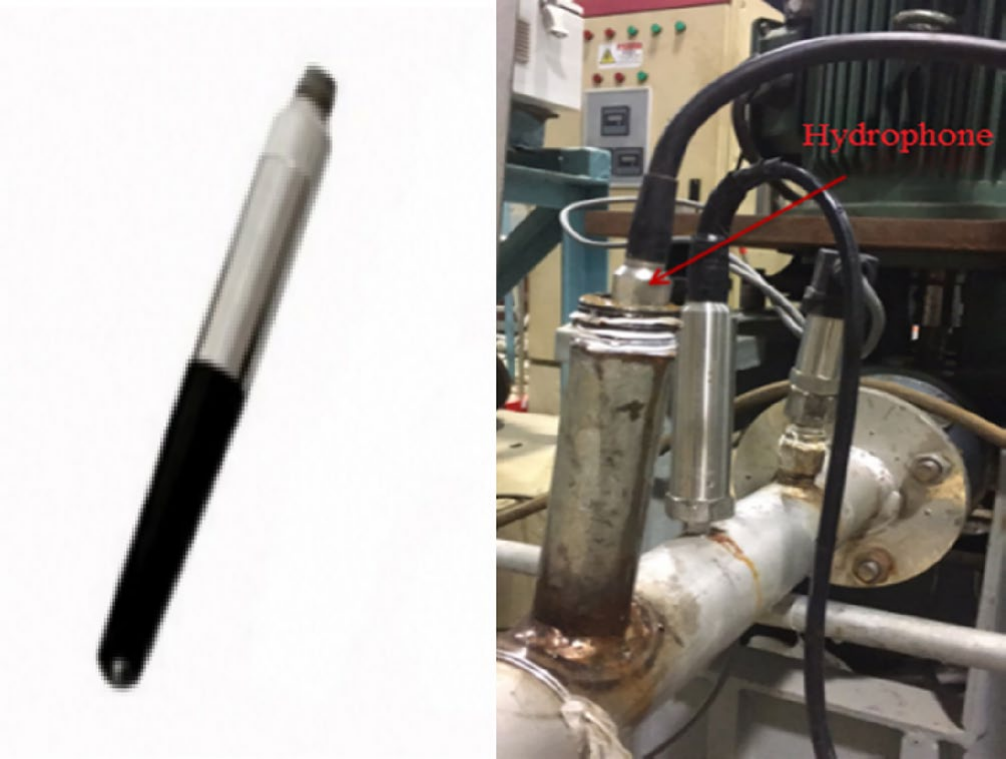
Hydrophone and installation location.

This paper uses the RHSA-10 hydrophone to measure the noise signal of marine pumps under rated operating conditions. The hydrophone has an operating frequency range of 20 Hz to 200 kHz. The sound pressure sensitivity is − 210 dB, the horizontal and vertical directivities are ± 2 dB and ± 2.5 dB, respectively, and the housing material is stainless steel. We install the hydrophone vertically at six times the pipe diameter of the pump inlet flange. This installation method can effectively measure the dipole sound source caused by the fluid pulsation in the pump, avoid the interference of other sound sources, and at the same time have little effect on the fluid flow in the internal field. The hydrophone and its installation location are shown in Fig. 10.

**Figure 11:**
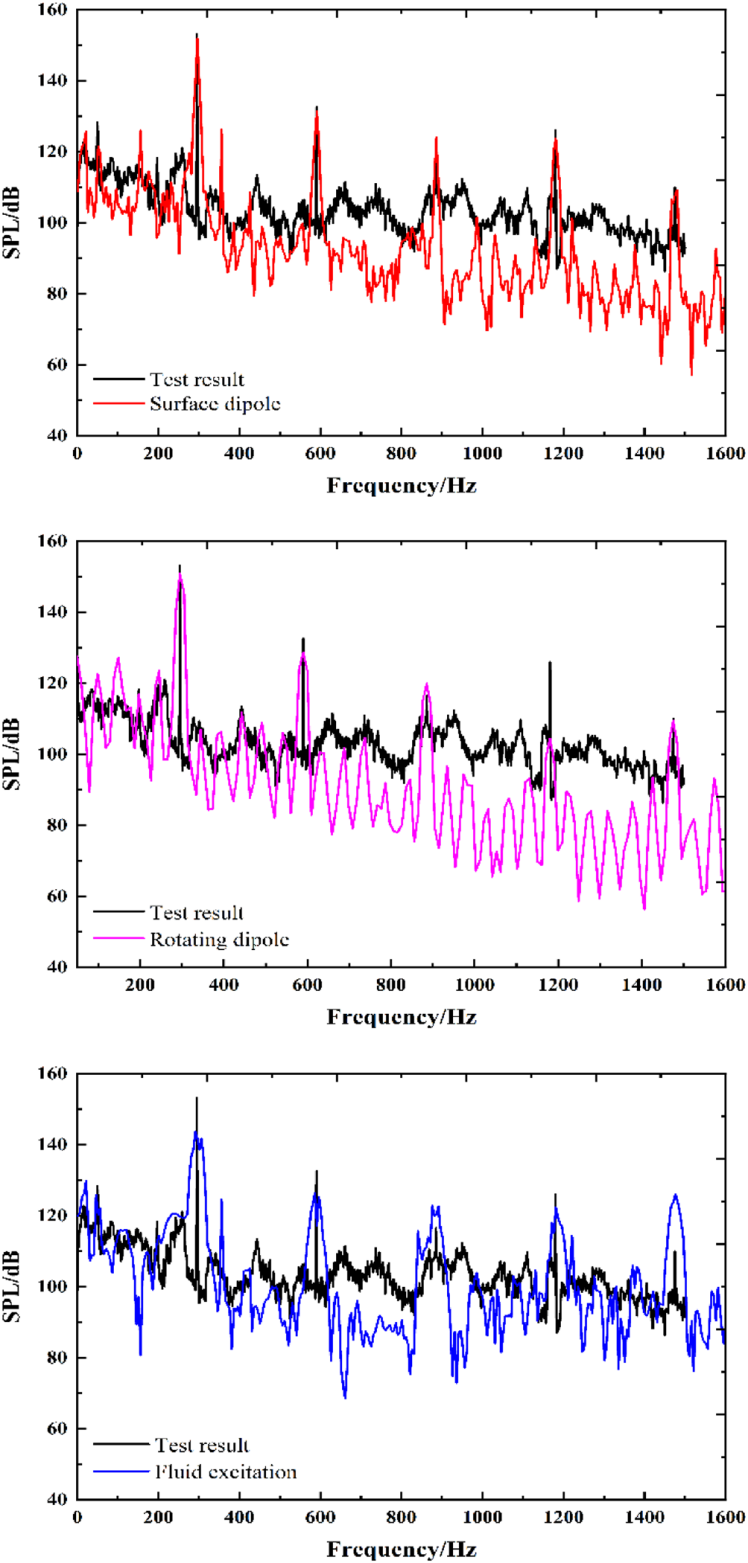
Broadband spectrum of the simulation and experimental results.

Figure 11 shows the comparison of the simulation and test results for the broadband spectrum at the outlet. The amplitude of each excitation source noise at the characteristic frequency generally agrees with the test results, and the simulation results for the surface dipole are closest. The results for three excitation sources show a decreasing trend throughout the frequency band. Moreover, this also indicates that it is feasible to predict internal fluid noise considering the coupled acoustic vibration. However, the test result is higher than the simulated value over the entire broadband. The reasons for this error are as follows:

1. There are interferences from other factors in the test process, such as valve noise, pipeline resonance and mechanical structural vibration, affecting the infield noise test results.
2. The sound waves inside the pump interact with the complex internal structure, resulting in reflection and scattering of the sound wave. There is also loss when the sound waves propagate. Moreover, the reflection and scattering of sound waves are ignored, which causes errors in the sound pressure level.

To clearly compare the contribution of different excitation sources to the noise of the marine pump units, the total sound pressure level *L*_p_ is introduced. The formula is as follows:

2 $$ L_{{\text{p}}} = 10{\text{log}}\int_{{f_{0} }}^{{f_{{{\text{max}}}} }} {\frac{{\left( {p_{{\text{a}}} /\sqrt 2 } \right)^{2} }}{{p^{2}_{0} }}} {\text{d}}f = 10\log \sum\limits_{{i = f_{0} }}^{{f_{\max } }} {\frac{{\left( {p_{i} /\sqrt 2 } \right)^{2} }}{{p^{2}_{0} }}} \Delta f_{i} $$

where Δ*f*_i_ is the minimum resolution. *f*_0_ and *f*_max_ are the initial and limit values of the calculated frequency, respectively, *p*_a_ and *p*_i_ are the root mean square (RMS) of the sound pressure, Pa, and *P*_0_ is the reference sound pressure, generally *P*_0_ = 1 × 10^–6^ Pa in water, and *P*_0_ = 2 × 10^–5^ Pa in air.

Table 3 shows the sound pressure levels of different excitation sources at the characteristic frequencies and compares the total sound pressure level and the test value. The dipole sound source is the primary factor at the main frequency. The rotating dipole source makes the dominant contribution at the characteristic frequencies, while the total sound pressure level is low in the frequency band, which highlights the “monophonic” feature of the rotating dipole source. The surface dipole contributes the most to the internal field noise of the pump, followed by the rotating dipole and then the fluid excitation. Compared with the test values, the total sound pressure level errors of the surface dipole, rotating dipole and fluid excitation are 1.1%, 1.25%, and 1.4%, respectively.

**Table 3:** Sound pressure levels for infield noise at characteristic frequencies.

Excitation sources	Frequency/Hz			
	BPF (295)	2BPF (590)	3BPF (885)	L_p_
Test value/dB	153.2	132.6	116.5	182.7
Fluid excitation/dB	144.0	126.0	122.6	180.2
Surface dipole/dB	149.8	128.0	123.0	180.6
Rotating dipole/dB	150.0	125.0	120.0	180.4

## External field noise

### Acoustic calculation of the external sound field

**Figure 12:**
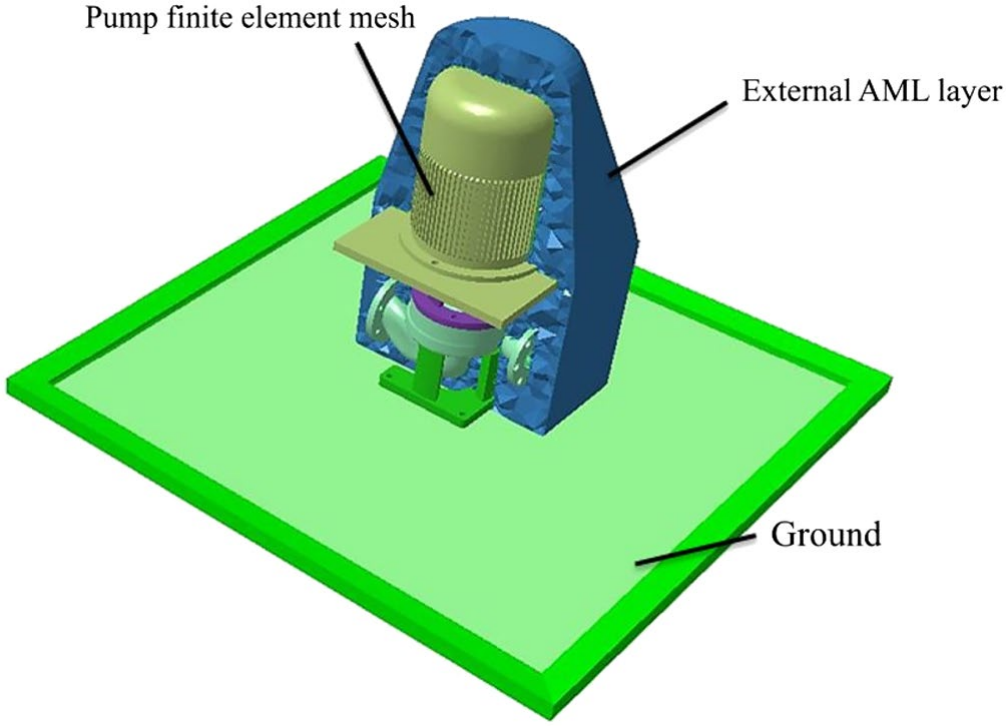
External sound field model.

According to the method of fluid-induced noise, the external field noise of marine pumps mainly includes the following: (1) The vibration and noise generated by the internal fluid directly acting on the pump. (2) The sound pressure of the dipole acoustic source acting on the pump structure, causing the structure to vibrate and radiate noise to the external space. The excitation is the same as the internal field noise, but the sound waves propagate in a different direction and form. In addition, the internal and external field mediums of marine pumps are different. The internal medium of the pump is water, and the external medium is air; therefore, the FEM/AML sound-vibration coupling method is used to calculate the external field noise. Figure 12 shows the external sound field model.

**Figure 13:**
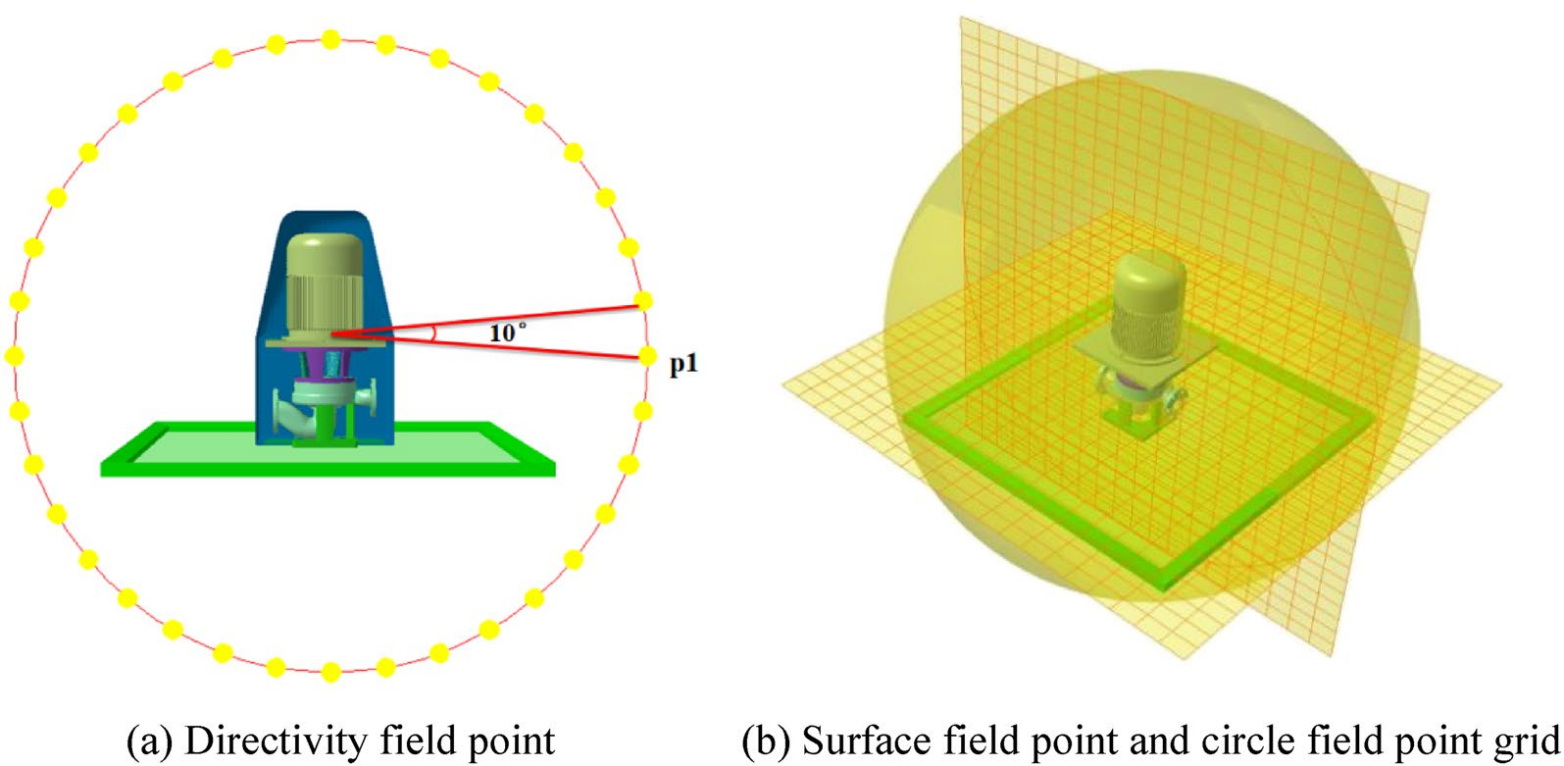
External sound field grid.

Inside the AML is the finite element model of the structure. The first layer of the AML mesh fits tightly with the outer surface of the internal structure shell, and a symmetrical surface is used as the ground. Sound propagation has pronounced directivity, and the sound pressure levels measured at different positions are also different. To obtain the sound pressure level distribution in the external field of the marine pump units, radial and axial monitoring surfaces are established with the model coordinate origin. Thirty-six monitoring points are set at 1 m from the coordinate origin, and the angle between each monitoring point is 10° to analyze the directivity distribution of external field noise. As shown in Fig. 13, a monitoring point p_1_ is set up to analyze the frequency response curve of the external field sound pressure.

### External field noise by fluid excitation

**Figure 14:**
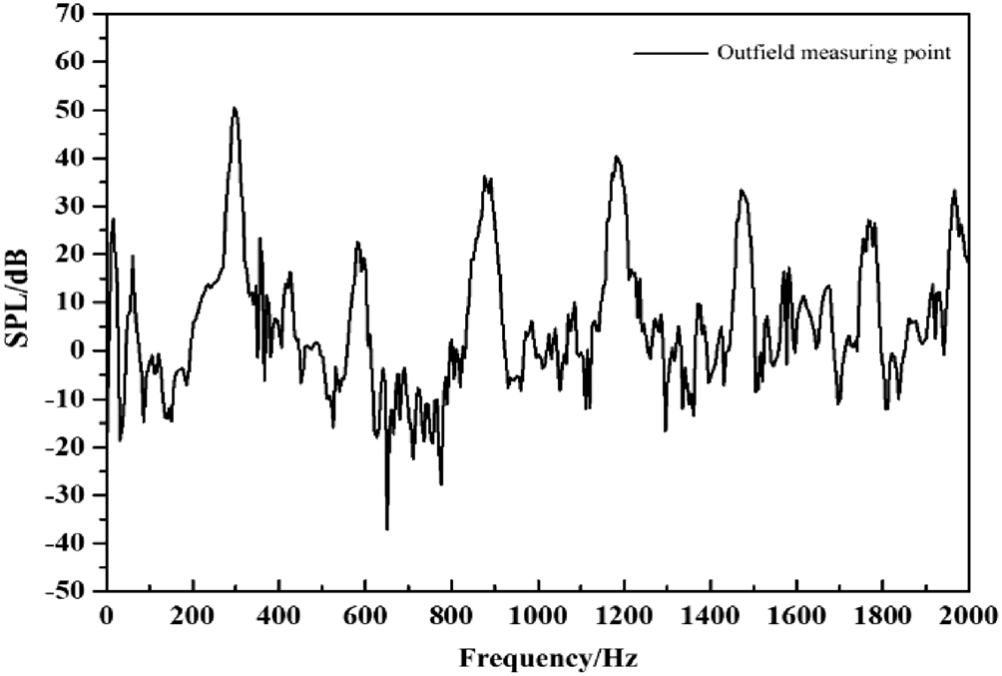
The spectrum of outfield noise by fluid excitation.

The external field noise induced by fluid excitation directly acts on the internal surface of the pump, causing vibration and noise. Figure 14 shows the frequency response curve of the fluid excitation. The main frequency of the external field noise is at the blade passing frequency, with a peak of 51.3 dB. The harmonic frequencies are located at the multiples. The second main frequency appears at 4BPF (1180 Hz), peaking at 40 dB.

**Figure 15:**
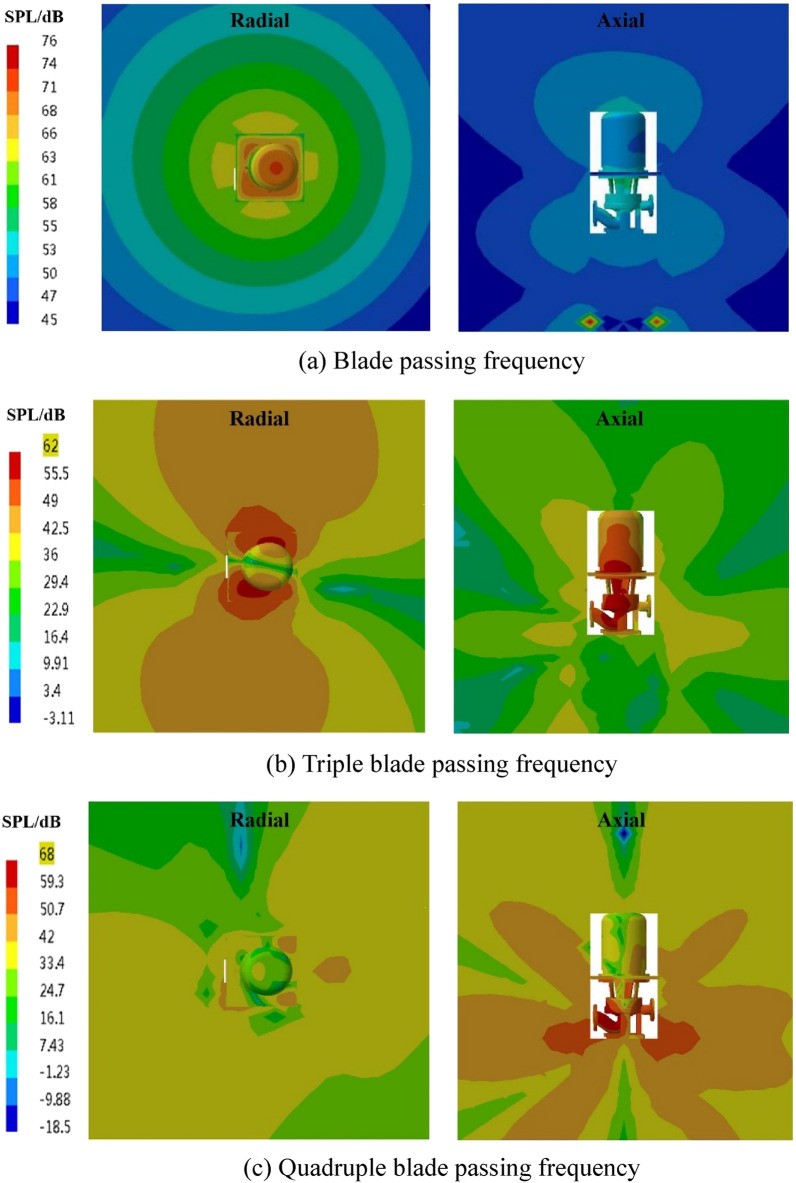
Distribution of outfield noise by fluid excitation.

**Figure 16:**
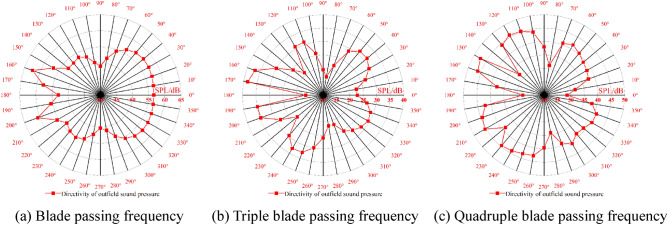
Directivity distribution of outfield noise by fluid excitation.

The spatial distribution of the external field radiated noise by the fluid excitation at the characteristic frequency is shown in Fig. 15. The inlet and outlet water pipes significantly influence the sound field distribution around the marine pump. The radial plane sound pressure at the BPF is distributed from the inlet and outlet water pipes, similar to a water wave shape, radiating uniformly outwards. The sound pressure level gradient decreases uniformly. The sound pressure distributions for 3BPF and 4BPF on the axial plane are similar, and the inlet and outlet sound pressure levels are relatively high. The sound pressure level at the outlet pipe reaches 62 dB at 3BPF and 68 dB at 4BPF. The noise generated by fluid excitation has different sound pressure levels at different characteristic frequencies and causes different acoustic vibration coupling patterns, so its sound pressure distribution is also different. Figure 16 shows that the directivity distribution of different frequencies is not the same, and the external field sound pressure at each frequency is not a regular circle. The external field noise interacts with the structural vibration and radiates to the far field, which makes the surrounding sound pressure distribution irregular.

### External field noise by surface dipole

**Figure 17:**
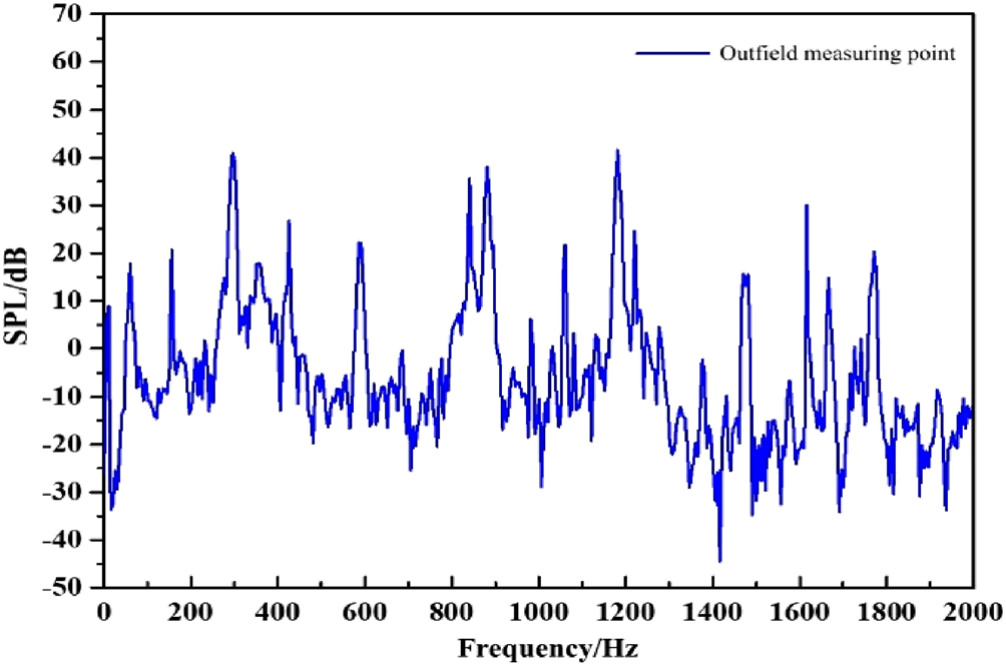
The spectrum of outfield noise by the surface dipole.

Figure 17 shows the frequency response curve of the sound pressure level by the surface dipole. The sound pressure generated by the surface dipole acts on the structure wall, causing structural vibration and radiating noise outwards. The main frequency of the radiation noise is at the blade passing frequency, with a peak of 41 dB. The second main frequency appears at the quadruple blade passing frequency of 1180 Hz, and the peak is 39 dB. The characteristics of each harmonic frequency are pronounced. Because of the influence of the structure’s natural frequency, there are many other peaks at the resonance frequency.

**Figure 18:**
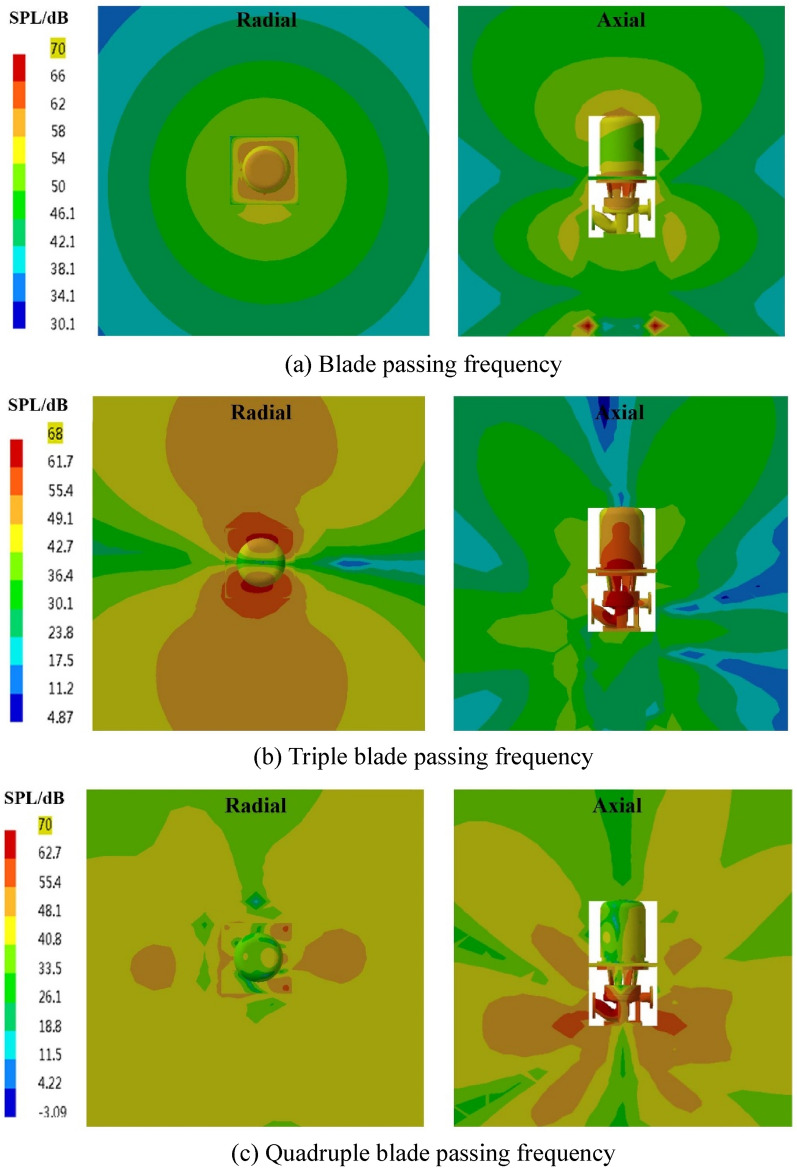
Pressure distribution of outfield noise by the surface dipole.

**Figure 19:**
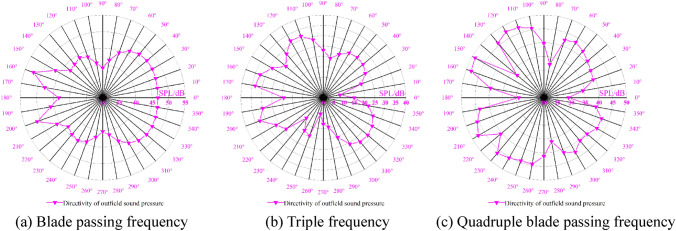
Directivity distribution of outfield noise by the surface dipole.

The spatial and directivity distributions of the external field noise generated by the surface dipole at the characteristic frequency are shown in Figs. 18 and 19. The spatial and directivity distributions of the external field noise caused by the surface dipole are very similar to those induced by fluid excitation at the characteristic frequency. However, the sound pressure levels by the surface dipole at each monitoring point are on average 10 dB less than the fluid excitation at the BPF. At 3BPF and the 4BPF, the difference between the sound pressure levels at each monitoring point for surface dipole and fluid excitation is small. This shows that the characteristic frequencies and pump structure play dominant roles in the spatial distribution of the radiated noise in the external field. Moreover, the different excitation sources mainly determine the size of the sound field.

### External field noise by the rotating dipole

**Figure 20:**
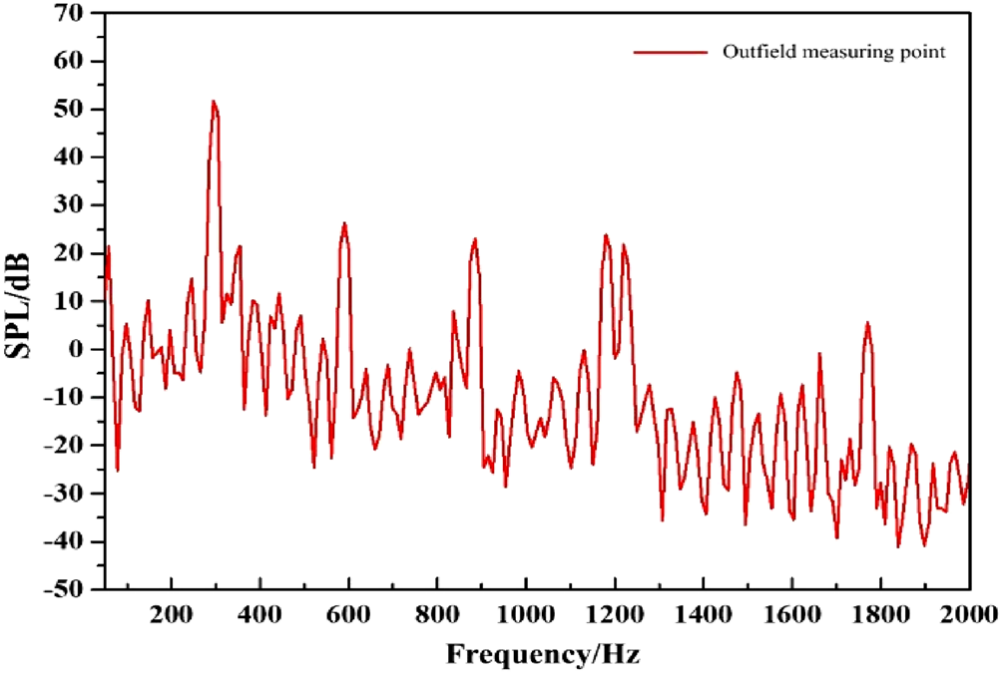
The spectrum of outfield noise by the rotating dipole.

Figure 20 shows the frequency response curve of the external field sound pressure level induced by the rotating dipole. The main frequency of the external field noise by the rotating dipole is at the BPF, and the harmonic frequencies are distributed at each multiple of the BPF. The maximum peak is at the BPF of 51 dB, and the sound pressure level decreases as the frequency rises. Moreover, the external and internal field noise spectra induced by the rotating dipole have similar features. The sound pressure level at the characteristic frequency is particularly pronounced and lower at other frequencies. This also indicates that the noise induced by the rotating dipole has the characteristics of a single tone, and its contribution is reflected only at the characteristic frequencies.

**Figure 21:**
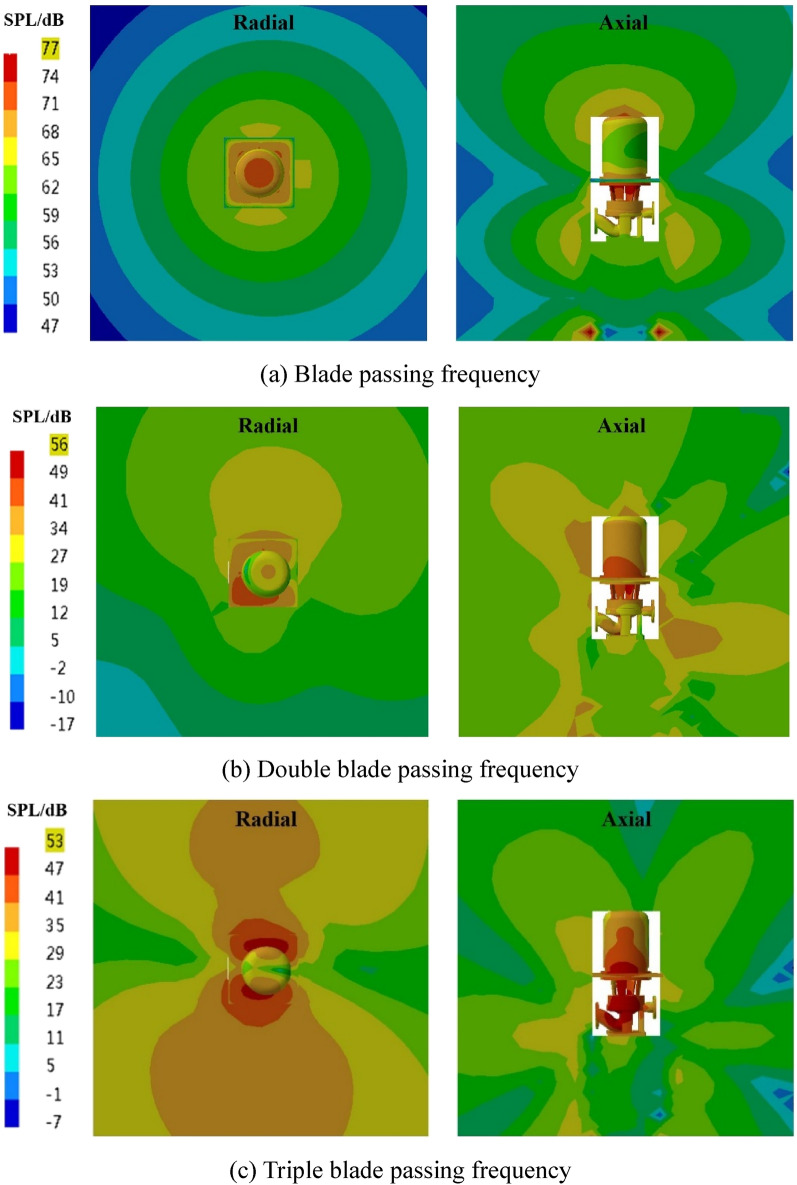
Pressure distribution of outfield noise by the rotating dipole.

**Figure 22:**
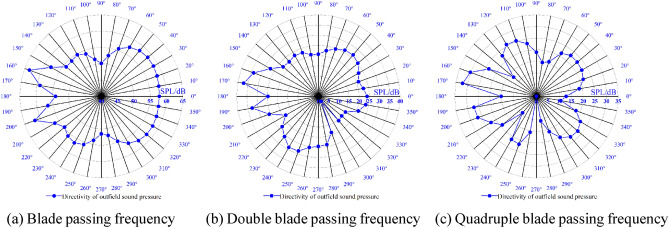
Directional distribution of outfield noise by the rotating dipole.

The spatial and directional distributions of the external field noise generated by the rotating dipole at the characteristic frequency are shown in Figs. 21 and 22. The sound pressure level induced by the rotating dipole at each monitoring point is slightly higher than the fluid excitation at the BPF. At 3BPF and the 4BPF, the sound pressure is significantly lower than the fluid excitation. The spatial distribution pattern of the external field radiation noise induced by different excitations is almost identical. However, the external field noise by fluid excitation is the largest in the far field. Due to the pump vibration generated by the fluid excitation being the most severe compared to the acoustic excitation, the external field noise generated by the fluid excitation dominates. However, the vibration features of the pump generated by the three excitation sources are similar. Therefore, the spatial distribution of noise at each characteristic frequency is also consistent.

**Figure 23:**
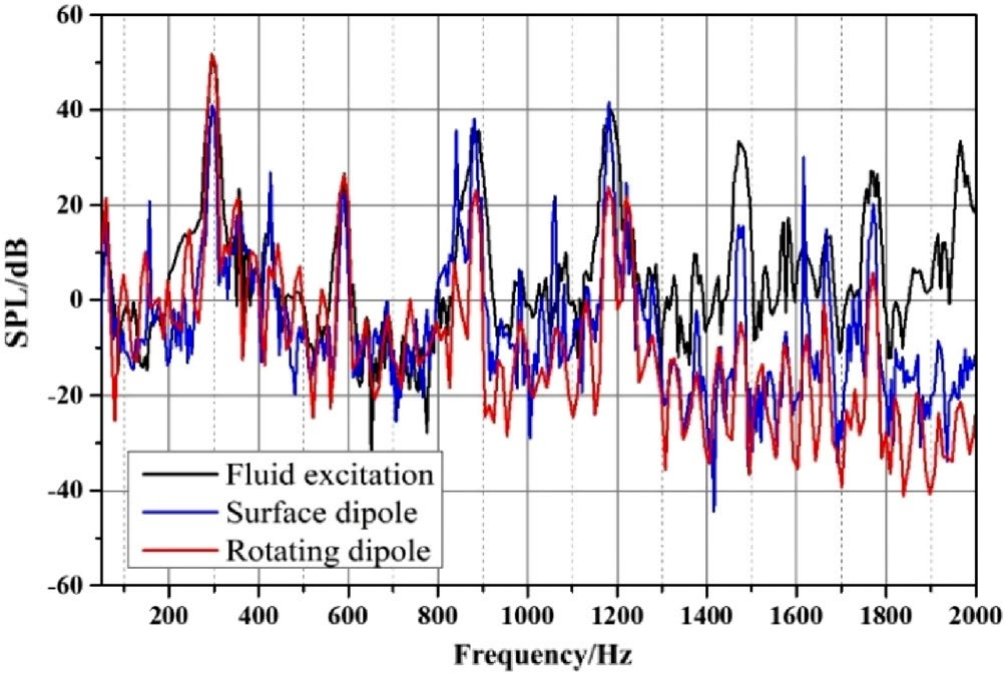
Broadband spectrum of outfield noise by different excitations.

Figure 23 compares the frequency response curves of the external field sound pressure generated by different excitations. The external field noise induced by different excitations is basically the same, and the main frequency is the blade passing frequency, followed by the quadruple blade passing frequency. Below 1200 Hz, the proportion of each excitation source is relatively consistent. However, the external field noise by fluid excitation is dominant between 1200 and 2000 Hz. At the main frequency, the sound pressure level induced by the fluid excitation and the rotating dipole has a better contribution, and the surface dipole accounts for a smaller proportion.

The sound pressure level at characteristic frequencies and the total sound pressure level are used to intuitively evaluate the contribution rate of different excitations to the external field noise, as shown in Table 4. At the BPF and at 2BPF, the fluid excitation and the rotating dipole contribute significantly to the external field noise of the marine pump. The corresponding sound pressure levels at the main frequency are 51.3 dB and 51.6 dB. The lowest is the surface dipole at 41 dB. At 3BPF and 4BPF, the sound pressure level induced by the fluid excitation is prominent. Moreover, the total sound pressure level of fluid excitation is higher than that of dipole excitation. Therefore, the external field noise induced by fluid excitation dominates the external field noise of marine pumps.

**Table 4:** Sound pressure levels for outfield noise at characteristic frequencies.

Excitation sources	Frequency/Hz			
	BPF (295)	2BPF (590)	3BPF (885)	L_p_
Fluid excitation/dB	51.3	26.6	38.0	139.2
Surface dipole/dB	41.0	22.1	36.0	136.3
Rotating dipole/dB	51.6	26.3	23.1	137.3

## Conclusions

The acoustic finite element method is used to calculate the internal field noise by fluid excitation and acoustic excitations, and the external field noise by different excitation sources is calculated based on the AML method. The spectral characteristics of different excitation sources and the distribution of the radiated sound field are analyzed. The contributions of different excitation sources to the internal and external sound fields are revealed. The conclusions are as follows:

1. The main frequency of the internal field noise induced by different excitation sources is distributed at the BPF (295 Hz). The second main frequency is at 2BPF (590 Hz), and each characteristic frequency is distributed at each multiple of the BPF. At the main frequency, the internal field noise induced by the dipole occupies a dominant position. Above 3BPF (885 Hz), fluid excitation gradually dominates.
2. The broadband spectrum of the internal field noise induced by the surface dipole agrees with the test result. The total sound pressure level errors of the surface dipole, rotating dipole and fluid excitation are 1.1%, 1.25%, and 1.4%, respectively. The contribution to the total sound pressure level of the infield noise is ordered as follows: surface dipole excitation (180.6 dB) > rotating dipole excitation (180.4 dB) > fluid excitation (180.2 dB).
3. The main frequency of the external field noise is the blade passing frequency, where the rotating dipole excitation contributes the most at 51.6 dB, and the lowest is the surface dipole at 41 dB. The external field noise induced by fluid excitation is dominant, especially between 1200 and 2000 Hz. Moreover, the external sound field distribution under different excitations is generally the same. The external field's total sound pressure level contribution is ordered as follows: fluid excitation (129.2 dB) > rotating dipole excitation (137.3 dB) > surface dipole excitation (136.3 dB).
